# Numerical and Experimental Determination of the Effective Mechanical Characteristics of a Polymer–Grease Contact Pair

**DOI:** 10.3390/polym18172083

**Published:** 2026-08-27

**Authors:** Anna A. Kamenskikh, Yuriy O. Nosov, Andrey R. Muhametshin

**Affiliations:** 1 Department of Computational Mathematics, Mechanics and Biomechanics, Perm National Research Polytechnic University, 614990 Perm, Russia; ura.4132@yandex.ru (Y.O.N.); muhametshin_science@mail.ru (A.R.M.); 2 Laboratory of Digital Engineering of Mechanical Engineering Processes and Production, Perm National Research Polytechnic University, 614990 Perm, Russia

**Keywords:** equivalent continuous medium, modified Anand model, elastic-viscous plasticity, effective characteristics, finite element method, ultrahigh-molecular-weight polyethylene, grease

## Abstract

Contact polymer–grease pairs of materials are widely used in highly loaded friction units. However, modeling the joint mechanical deformation of polymer grease significantly increases computational costs and complicates the convergence of the numerical solution. This is due to the pronounced nonlinear behavior of the materials and the significant difference in their physical and mechanical characteristics. Modeling the behavior of complex spatial configurations of structures, taking into account temperature and time factors, and investigating the behavior of structures in dynamics challenges researchers and engineers with the task of reducing computational costs for numerical experiments without loss of accuracy. Therefore, it is of great importance to design methods and approaches for describing effective characteristics, taking into account the nonlinear behavior of a polymer–grease pair of materials. Representing the design volumes of polymer grease as an equivalent medium can reduce computational costs. This paper considers the design volume, including a polymer base made of ultrahigh-molecular-weight polyethylene and a spherical recess filled with CIATIM-221 grease. The modified elastic–viscoplastic Anand model has been used to describe the behavior of materials and an equivalent medium. The model parameters have been determined based on a set of numerical experiments at various temperatures, deformation rates, and sizes of the design volume. The effective elastic and viscoplastic characteristics of the equivalent medium have been determined. An increase in temperature leads to a decrease in stiffness and resistance to viscoplastic flow. Increasing the size of the design volume helps to stabilize effective characteristics and reduce the impact of local structural heterogeneities. The proposed analytical dependencies make it possible to take into account the influence of temperature and the geometry of the design volume when determining the parameters of an equivalent medium. The developed approach can be used in finite element modeling of large structures with friction units to reduce computational costs without explicitly modeling the polymer–grease interface.

## 1. Introduction

Friction units are important elements of modern engineering structures [1,2]. They are widely used in facilities operating under high contact loads over the long term [3,4,5,6,7]: transport and logistics systems, aerospace engineering, mechanical engineering, etc. The durability of friction units is determined by physical–mechanical, tribological, and other properties of materials [5,8]. It is noted that 80% of cases of equipment downtime are associated with the failure of friction units [5]. This requires a numerical and experimental evaluation of the performance of materials and units.

Antifriction polymer and composite materials [9,10] in combination with greases [3,4,11] are used to reduce friction effects during the interaction of elements. Polymers/composites perceive the main mechanical load [12,13]. Greases reduce friction, reduce wear, and promote uniform distribution of contact stresses [14,15,16]. The joint deformation behavior of a pair of polymer–grease materials is an important factor when assessing the operability of the structure.

Progress in numerical methods has made it possible to study the mechanical behavior of friction units under various loading and operating conditions. The finite element method (FEM) is widely used to describe contact interaction, stress–strain state, temperature effects and time-dependent deformations [17,18,19,20]. Such approaches are particularly important for friction units, since their operational behavior is determined by the combined influence of material properties, contact conditions, temperature, and loading rate.

Computer engineering methods, including the FEM, are used in the modeling of friction units [17,18]. FEM makes it possible to evaluate the stress–strain state of the structure and its friction characteristics, temperature and time effects, as well as the structural parameters of the interface surfaces [19,20]. Computer engineering makes it possible to predict the life of a structure and investigate the influence of various operational factors at the design stage.

Polymer and composite materials suitable for use in friction units are widely studied. The most attention is paid to investigating physical–mechanical characteristics under different conditions [8,9,21,22]: change in the deformation rate; temperature change; relaxation tests; creep tests; frictional properties, etc. It has been experimentally shown that this class of materials has nonlinear properties, exhibiting both viscoelastic and elastic–viscoplastic responses [8,9]. Polytetrafluoroethylene (PTFE) is widely used among antifriction materials. PTFE has good antifriction properties under high loads, heat resistance, etc. [16]. However, the material has a number of disadvantages [23], including high wear rate and low yield strength. Ultrahigh-molecular-weight polyethylene (UHMWPE) is an alternative to PTFE for highly loaded friction units [24]. UHMWPE has higher physical and mechanical characteristics in the operating temperature range from −200 to +100 °C. This allows the material to be used in many structures.

Grease lubricants are widely used in critical friction units during long periods of operation [25,26]. These are multicomponent systems consisting of base oil, thickener, and functional additives [27,28,29]. A different combination of components and their percentage composition leads to a wide variability of physical–mechanical, tribological, thermophysical, and other properties of the final material [30,31,32]. For example, metal oxide nanoparticles CEO_2_ and CuO demonstrate unique interfacial interactions and effects when embedded in the lubricant structure compared to traditional additives [33]. The introduction of Fe_3_O_4_/WS_2_ and CuO/WS_2_ lubricants into the lubricant reduces the likelihood of spark discharge in the bearings of electric machines [34]. Polymer additives, due to their basic characteristics, make it possible to increase the physical–mechanical and tribological properties of greases [35].

The experimental research framework is developed together with the creation of mathematical models for describing materials. The use of the relations of the theory of viscoelasticity with a number of assumptions is accepted to describe the behavior of both polymer and grease lubricants [36,37,38,39]. Different theories describing the behavior of a material as part of a static and quasi-static problem were also described [9]. The results show that nonlinear theories are effective for the qualitative description of the behavior of materials. Using ratios of theories of elastic–viscous plasticity can account for a wider range of operating conditions [40].

A small number of papers on the joint deformation of a pair of polymer–grease materials can be noted. Research on polymer/composite materials and greases separately from each other is currently prevalent. This approach can accurately describe the behavior of a material. However, it does not reflect the features of the polymer–grease system working together. This results in a significant deviation of the numerical order of the friction units from the actual design. Component-by-component modeling of materials in a large-sized structure leads to a significant increase in computational costs. This problem arises due to significant differences in the physical and mechanical characteristics of individual elements. For example, the modulus of elasticity of polymers/composites differs from the modulus of elasticity of greases by several orders of magnitude [9,24]. This, in turn, leads to a deterioration in the conditionality of the system of linear algebraic equations. This together reduces the effectiveness of computer engineering to rationalize the behavior of the structure.

One of the effective ways to reduce computational costs is the use of homogenization methods [41]. The main idea is to replace an inhomogeneous structure with an equivalent continuous medium with effective mechanical characteristics and reproducing the integral mechanical response of the original system [42]. This approach is widely used in the study of composite materials, multilayer structures, and porous media. The polymer–grease contact pair differs significantly from the usual composite materials. Polymer materials and greases exhibit pronounced nonlinear behavior at different deformation rates and temperatures. The differences between the properties can reach more than 80%. Standard methods for determining effective characteristics are limited and cannot be used to describe the polymer–grease system.

This article is intended to form methods and approaches for describing the nonlinear behavior of the polymer–grease system as an equivalent continuous medium. This is important for the numerical modeling of complex spatial configurations of structures under a wide range of temperature and force influences, including those with limited computing resources.

The hypothesis of joint polymer–grease mechanical behavior in the form of a design volume was introduced as part of the research. The design volume can be represented as an equivalent continuous medium with effective characteristics determined based on a numerical and experimental approach. The research is aimed at a mathematical description of an equivalent continuous medium in the form of a design volume using the examples of UHMWPE and CIATIM-221 grease.

## 2. Materials and Methods

### 2.1. Design Volume

**Figure 1:**
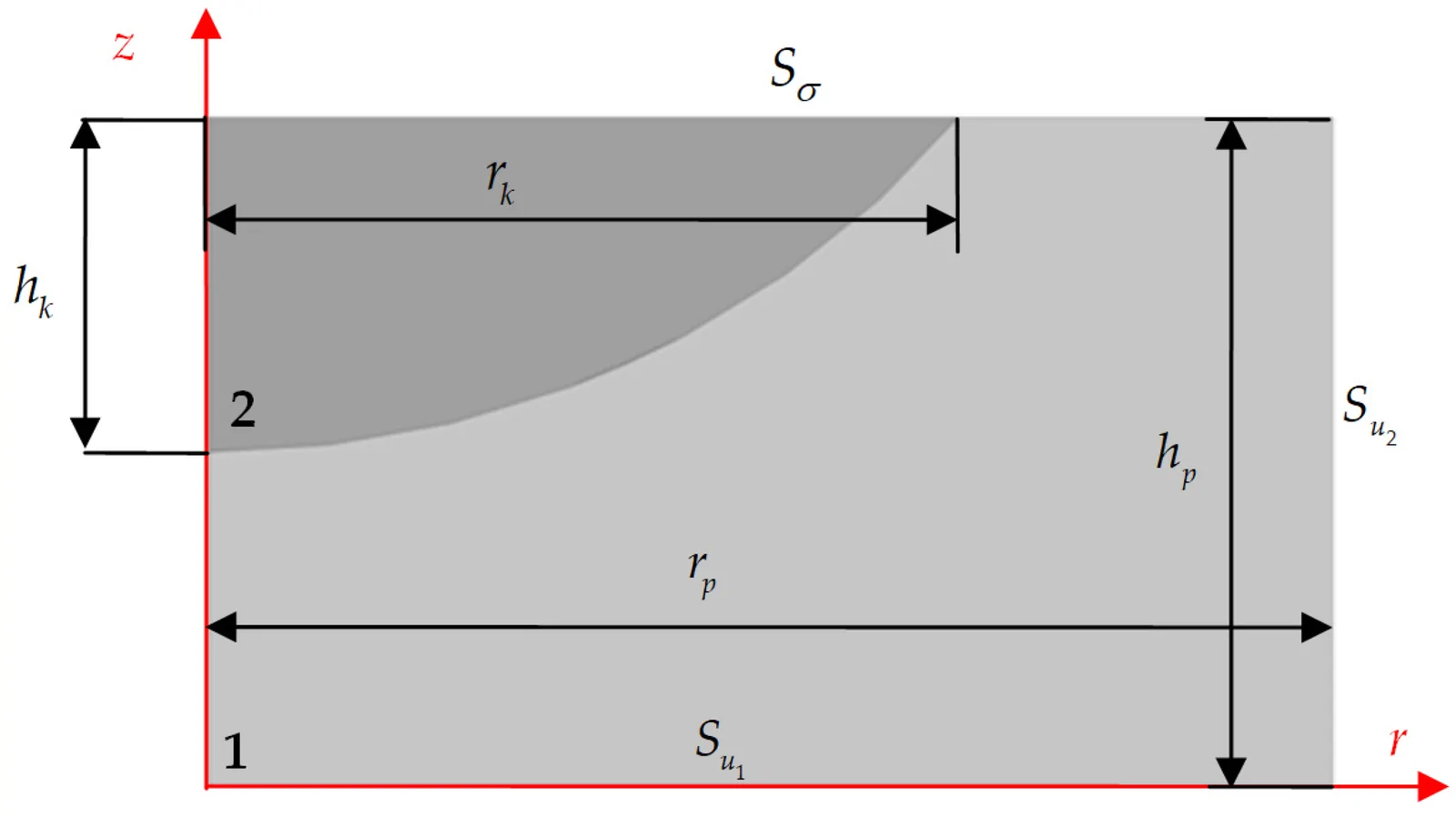
Geometry of the design volume: 1 is polymer; 2 is grease.

A design volume with one recess for the grease in the form of a spherical cutout was considered (Figure 1). This type of polymer–grease system is not suitable for the standard homogenization method. A numerical–experimental approach is necessary to find effective characteristics of an equivalent medium. The approach involves a comprehensive experimental study of the design volume of materials and numerical interpretation using computer engineering methods.

Geometric configuration of the design volume: $h_{k}$ = 2 mm is the depth of the recess for grease; $d_{k}=2r_{k}$ = 8 mm is the diameter of the recess for grease; $h_{p}$ = 4 mm is the thickness of the interlayer; $d_{p}=2r_{p}$ = 12 to 28 mm is the diameter of the rated volume; $S_{\sigma }$ is load application surface; $S_{u_{1}}$ and $S_{u_{2}}$ are surfaces with given displacement constraints; and *r* and *z* are radial and axial direction, respectively. The diameter of the design volume acts as a variable parameter. This allows us to consider various schemes for filling the antifriction layer with recesses for the grease.

The diameter and depth of the grease recess were constant: $d_{k}$ = 8 mm and $h_{k}$ = 2 mm. The diameter of the design volume varied from 12 to 28 mm. The volume fraction of the grease varied with the calculated volume size. The volume fraction of the grease was 12, 4.3 and 2.2% for $d_{p}$ = 12, 20 and 28 mm, respectively. The dependence of the effective characteristics on $d_{p}$ should be interpreted as the combined effect of the design volume size and the relative grease volume fraction.

The polymer and grease exhibit nonlinear behavior depending on temperature, time, and rate of deformation [43,44]. The theory of elasto-viscoplasticity is chosen to describe nonlinear behavior. The digital equivalent of the materials is based on the constitutive model of Anand.

The experimental data obtained in previous papers [45,46] were used to form models of the behavior of materials of the design volume and equivalent medium. Design volume 1 was UHMWPE (AlfaTech LLC, Perm, Russia); 2 was CIATIM-221 (Central Institute of Aviation Fuels and Oils, Moscow, Russia).

The model of joint deformation of a polymer–grease pair of materials is considered taking into account the ideal contact at the polymer–grease interface.

To determine the pattern of the stress–strain state, forces are applied to the boundary $S_{\sigma }$ at different rates. The loading rate corresponds to the rate of deformation of the combined material: 2.5%/min; 25%/min; 50%/min; 100%/min. This helps describe the behavior of materials at different rates of deformation.

### 2.2. Determination of Elastic Parameters of an Equivalent Medium

The elastic characteristics of the equivalent medium must be determined as part of the first stage of the research. The problem is solved in a static formulation to determine the elastic characteristics.

The mathematical formulation of the problem includes the equations of equilibrium:

(1) divσ=0.

Small loads are applied to determine the elastic characteristics. Therefore, the Cauchy relation has the form

(2) $$\varepsilon =\frac{1}{2}\left(\nabla \;\bar{u}+{\left(\nabla \;\bar{u}\right)}^{T}\right),$$

where $\bar{u}$ is the displacement vector.

The relationship between the stress tensor and the deformation tensor has the form

(3) σ=C:ε,

where **C** is the matrix of elastic constants.

Young’s modulus is determined from the relation in the case of a uniaxially stressed state:

(4) $$\sigma_{z}=E\varepsilon_{z}.$$

The modulus of constrained compression M is determined from the experiment for a uniaxial deformed state:

(5) $$\sigma_{z}=M\varepsilon_{z}.$$

Poisson’s ratio is found through the relationship between Young’s modulus *E* and the constrained compression modulus *M*:

(6) $$\nu =\frac{4}{\left(1+3M/E-\sqrt{{\left(1+3M/E\right)}^{2}-16M/E}\right)}-1.$$

The spectrum of relaxation processes allows the description of properties in the form of a finite set of exponentials [47]. The dependencies of Young’s modulus *E* and Poisson’s ratio ν on the temperature and diameter of the design volume were introduced and have the form

(7) $$E\left(T,d_{p}\right)=E_{1}\left(d_{p}\right)\times \left[1+E_{2}\left(d_{p}\right)\times e^{\left(\frac{E_{3}\left(d_{p}\right)}{T}\right)}\right]\times \left[1+E_{4}\left(d_{p}\right)\times e^{\left(\frac{E_{5}\left(d_{p}\right)}{T}\right)}\right],$$

(8) $$\nu \left(T,d_{p}\right)=\nu_{1}\left(d_{p}\right)\times \left[1+\nu_{2}\left(d_{p}\right)\times e^{\left(\frac{\nu_{3}\left(d_{p}\right)}{T}\right)}\right]\times \left[1+\nu_{4}\left(d_{p}\right)\times e^{\left(\frac{\nu_{5}\left(d_{p}\right)}{T}\right)}\right],$$

where the functional dependencies of Young’s modulus and Poisson’s ratio on the diameter of the design volume have the form

(9) $$E_{i}\left(d_{p}\right)=E_{i1}\times \left[1+E_{i2}\times e^{E_{i3}\times d_{p}}\right]\times \left[1+E_{i4}\times e^{E_{i5}\times d_{p}}\right],$$

(10) $$\nu_{i}\left(d_{p}\right)=\nu_{i1}\times \left[1+\nu_{i2}\times e^{\nu_{i3}\times d_{p}}\right]\times \left[1+\nu_{i4}\times e^{\nu_{i5}\times d_{p}}\right],$$

where *i* takes a value from 1 to 5.

Similar parameters need to be found to describe the nonlinear behavior of materials by the constitutive Anand model.

### 2.3. Constitutive Model of Viscoplastic Behavior

Viscoelastic constitutive models allow a more detailed description of time-dependent reversible deformation, especially during relaxation, creep, unloading and cyclic loading. Such approaches are successfully applied to polymeric materials and greases [36,37,38,39]. However, the present study is aimed at monotonic loading and determination of effective elastic-viscoplastic characteristics in the range of temperatures and strain rates. A separate viscoelastic component was not introduced into the current homogenization procedure. The choice of the Anand model is related to the specific goal of determining the effective strain rate- and temperature-dependent viscoplastic characteristics of the polymer–grease system under monotonic deformation. The model provides a continuous description of inelastic deformation and takes into account strain rate sensitivity, temperature dependence of deformation resistance, activation energy and hardening/softening effects. These features are important for the polymer–lubricant system under consideration, since its stress–strain diagrams depend on both temperature and strain rate.

The constitutive Anand model is widely used in the mechanics of deformable solids. The model does not take into account the crystal structure of UHMWPE; the material is considered as an amorphous body. The classical Anand model was previously widely used to describe the behavior of a metal above the recrystallization temperature [48,49]. However, it is currently actively used to describe polymers, glass, lubricants, and other non-metallic materials [44]. The special feature of the Anand model is its continuous flow under any load. The greater the load, the higher the velocity, which is observed in amorphous materials.

The rate of viscoplastic deformation is described by the flow equation of the following form:

(11) $${\dot{\varepsilon }}^{vp}={\dot{\varepsilon }}_{eqv}^{vp}\left(3D_{\sigma }/2\sigma_{eqv}\right),$$

where ${\dot{\varepsilon }}^{vp}$ is the tensor of the rate of viscoplastic deformation; ${\varepsilon ˙}_{eqv}^{vp}=\sqrt{2/3\left({\dot{\varepsilon }}^{vp}:{\dot{\varepsilon }}^{vp}\right)}$ is the equivalent rate of viscoplastic deformation; $D_{\sigma }=\sigma -\sigma_{avg}I$ is the stress tensor deviator; σ is the stress tensor; I is the unit tensor; $\sigma_{avg}=\sum_{i=1}^{3}\sigma_{ii}/3$ is the average stress; and $\sigma_{eqv}=\sqrt{3/2\left(D_{\sigma }:D_{\sigma }\right)}$ is the equivalent stress.

The constitutive equation of the classical Anand model has the form

(12) $${\dot{\varepsilon }}_{eqv}^{vp}=\left\{\begin{array}{l} <<1,\;\mathrm{if}\;\sigma_{eqv}<s_{0}\;\\ Ae^{-U/RT}{\left[\sinh\left(\xi \left[\frac{\sigma_{eqv}}{s}\right]\right)\right]}^{1/m},\;\mathrm{if}\;\sigma_{eqv}\geq s_{0} \end{array}\right..$$

The model also includes an evolutionary equation for the relationship between deformation resistance and deformation rate:

(13) $$\dot{s}=\left\{h_{0}{\left(\left|B\right|\right)}^{a}\frac{B}{\left|B\right|}\right\}{\dot{\varepsilon }}_{eqv}^{vp},$$

(14) $$B=1-\frac{s}{s^{*}},$$

(15) $$s^{*}=\overset{⌢}{s}{\left[\frac{{\dot{\varepsilon }}_{eqv}^{vp}}{A}e^{\frac{U}{RT}}\right]}^{n},$$

where s is the deformation resistance [Pa]; $s_{0}$ is the initial value of deformation resistance [Pa]; *U* is the activation energy [kJ/mole]; *R* is the universal gas constant [$\mathrm{kJ}/\left(\mathrm{mole}\cdot K\right)$]; T is the temperature [K]; $h_{0}$ is the hardening/softening constant [Pa]; and $\overset{⌢}{s}$ is the coefficient for deformation resistance saturation value [Pa]. In the dimensionless form, A is the pre-exponential factor; *m* is the strain rate sensitivity of stress; *a* is the strain rate sensitivity of hardening or softening; ξ is the multiplier of stress; and *n* is the strain rate sensitivity of saturation (deformation resistance) value.

The modified Anand model, in which a number of parameters depend on temperature, is used as part of the research: deformation resistance $s_{0}$, activation energy *U*, and strain rate sensitivity of stress m. Let us introduce the dependence of these parameters on temperature:

(16) $$s_{0}\left(T\right)=N_{1}\left(1+N_{2}e^{\left[\frac{N_{3}}{T}\right]}\right)\left(1+N_{4}e^{\left[\frac{N_{5}}{T}\right]}\right),$$

(17) $$U\left(T\right)=N_{6}\left(1+N_{7}e^{\left[\frac{N_{8}}{T}\right]}\right)\left(1+N_{9}e^{\left[\frac{N_{10}}{T}\right]}\right),$$

(18) $$m\left(T\right)=N_{11}\left(1+N_{12}e^{\left[\frac{N_{13}}{T}\right]}\right)\left(1+N_{14}e^{\left[\frac{N_{15}}{T}\right]}\right),$$

where $N_{1}-N_{15}$ are the coefficients of the functions (6)–(8). $N_{1},\;N_{6},\;N_{11}$ have a dimension consistent with the dimensions of the parameters. $N_{3},\;N_{5},\;N_{8},\;N_{10},\;N_{13},\;N_{15}$ have a temperature dimension of [K]. $N_{2},\;N_{4},\;N_{7},\;N_{9},\;N_{12},\;N_{14}$ are dimensionless.

The Arrhenius equation [50] is used to describe the temperature dependence of the viscosity of a material and to form a relationship with a number of parameters of the modified Anand model:

(19) $$\eta \left(T\right)=Le^{\frac{U\left(T\right)}{RT}},$$

where L=1/A is the proportionality coefficient, which is related to the parameter of the constitutive Anand model.

When searching for effective characteristics of an equivalent medium, it is necessary to take into account the diameter of the design volume (Section 2.1). The dependencies of the parameters of the constitutive Anand model on the diameter of the design volume for the ratios (16)–(18) were introduced:

(20) $$s_{0}\left(T,d_{p}\right)=s_{1}\left(d_{p}\right)\times \left[1+s_{2}\left(d_{p}\right)\times e^{\left(\frac{s_{3}\left(d_{p}\right)}{T}\right)}\right]\times \left[1+s_{4}\left(d_{p}\right)\times e^{\left(\frac{s_{5}\left(d_{p}\right)}{T}\right)}\right],$$

(21) $$U\left(T,d_{p}\right)=U_{1}\left(d_{p}\right)\times \left[1+U_{2}\left(d_{p}\right)\times e^{\left(\frac{U_{3}\left(d_{p}\right)}{T\;}\right)}\right]\times \left[1+U_{4}\left(d_{p}\right)\times e^{\left(\frac{U_{5}\left(d_{p}\right)}{T}\right)}\right],$$

(22) $$m\left(T,d_{p}\right)=m_{1}\left(d_{p}\right)\times \left[1+m_{2}\left(d_{p}\right)\times e^{\left(\frac{m_{3}\left(d_{p}\right)}{T}\right)}\right]\times \left[1+m_{4}\left(d_{p}\right)\times e^{\left(\frac{m_{5}\left(d_{p}\right)}{T}\right)}\right],$$

where the dependencies of parameters (20)–(22) on the diameter of the design volume take the form

(23) $$U_{i}\left(d_{p}\right)=U_{i1}\times \left[1+U_{i2}\times e^{U_{i3}\times d_{p}}\right]\left[1+U_{i4}\times e^{U_{i5}\times d_{p}}\right],$$

(24) $$m_{i}\left(d_{p}\right)=m_{i1}\times \left[1+m_{i2}\times e^{m_{i3}\times d_{p}}\right]\left[1+m_{i4}\times e^{m_{i5}\times d_{p}}\right]$$

(25) $$s_{i}\left(d_{p}\right)=s_{i1}\times \left[1+s_{i2}\times e^{s_{i3}\times d_{p}}\right]\left[1+s_{i4}\times e^{s_{i5}\times d_{p}}\right],$$

where i varies from 1 to 5.

Thus, Equations (20)–(22) are not independent defining laws. They represent empirical approximations of the identified parameter values. Temperature-dependent terms describe the change in the parameters of the constitutive model depending on temperature. Scale-dependent coefficients take into account the change in the parameters of the constitutive model depending on the heterogeneous structure’s characteristic size.

Dependencies of the form (23)–(25) are used to describe model parameters independent of temperature, which have the form

(26) $$n\left(d_{p}\right)=n_{1}\times \left[1+n_{2}\times e^{n_{3}\times d_{p}}\right]\times \left[1+n_{4}\times e^{n_{5}\times d_{p}}\right],$$

(27) $$\overset{⌢}{s}\left(d_{p}\right)={\overset{⌢}{s}}_{1}\times \left[1+{\overset{⌢}{s}}_{2}\times e^{{\overset{⌢}{s}}_{3}\times d_{p}}\right]\left[1+{\overset{⌢}{s}}_{4}\times e^{{\overset{⌢}{s}}_{5}\times d_{p}}\right],$$

(28) $$\xi \left(d_{p}\right)=\xi_{1}\times \left[1+\xi_{2}\times e^{\xi_{3}\times d_{p}}\right]\left[1+\xi_{4}\times e^{\xi_{5}\times d_{p}}\right],$$

(29) $$h_{0}\left(d_{p}\right)=h_{1}\times \left[1+h_{2}\times e^{h_{3}\times d_{p}}\right]\left[1+h_{4}\times e^{h_{5}\times d_{p}}\right],$$

(30) $$a\left(d_{p}\right)=a_{1}\times \left[1+a_{2}\times e^{a_{3}\times d_{p}}\right]\left[1+a_{4}\times e^{a_{5}\times d_{p}}\right].$$

The numerical procedure for searching for parameters of the constitutive model previously described in [44] was used to search for a vector of unknowns.

The stress state is characterized by the Cauchy stress tensor. The tensor is used to calculate the equivalent stress, which is included in the law of viscoplastic flow of the Anand model. The hypoelastic model is used to describe the reversible component of the deformation:

(31) $$\sigma =C:\varepsilon^{el}=C:\left(\varepsilon -\varepsilon^{vp}\right),$$

where $\varepsilon^{el}$ is the elastic deformation tensor; ε is the total deformation tensor; and $\varepsilon^{vp}$ is the viscoplastic deformation tensor. The hypoelastic ratio is written in terms of the objective derivative of the stress tensor and the strain rate in differential form. This makes it possible to relate the elastic component of the deformation to the law of viscoplastic flow of the Anand model:

(32) $$\hat{\sigma }=C:\left(\dot{\varepsilon }-{\dot{\varepsilon }}^{vp}\right),$$

where $\hat{\sigma }$ is the objective derivative of the stress tensor and $\dot{\varepsilon }$ is the rate tensor of total deformation.

The kinematic relations are written in terms of the Green–Lagrange finite deformation tensor using a geometrically nonlinear formulation of the problem:

(33) $$\varepsilon =\frac{1}{2}\left(\nabla \;\bar{u}+{\left(\nabla \;\bar{u}\right)}^{T}+\left({\left(\nabla \;\bar{u}\right)}^{T}\cdot \nabla \;\bar{u}\right)\right).$$

The presented system of equations defines a complete mathematical formulation of the problem, including a description of the elastic and viscoplastic behavior of the material under conditions of large deformations. The Anand constitutive model is implemented in the ANSYS Mechanical APDL 2021R2 software package (Livermore, CA, USA) using a geometrically nonlinear formulation. The resulting model was used to determine the effective characteristics of an equivalent medium and analyze the stress–strain state of the design volume under various temperature conditions and geometric parameters.

A finite element calculation of a heterogeneous model consisting of a polymer and grease region was initially performed to determine the polymer–grease effective characteristics. The Anand model parameters, given in Table 1 and Table 2, were specified for each region (material). Ideal contact of materials, ensuring compatibility of their deformation, was realized at the polymer–grease interface. The relationship between stresses and strains for the entire calculated volume was obtained as a result. The obtained dependencies were used to determine the effective characteristics and identify the Anand model parameters of an equivalent environment.

## 3. Results and Discussion

### 3.1. Obtaining Initial Parameters for Identification

The previously identified temperature-dependent parameters of the constitutive Anand model for UHMWPE and CIATIM-221 were used as the initial data for numerical modeling. The parameters were identified based on experimental data in the temperature range of 233–353 K. The obtained parameters describe the mechanical behavior of each material individually and are shown in Table 1 and Table 2.

The use of individual constitutive models of components makes it possible to reproduce the joint deformation of the polymer base and the grease within the design volume. The stress–strain diagrams obtained as a result of numerical modeling will be used in the future to determine the effective elastic characteristics and identify the parameters of the constitutive model of an equivalent medium.

Modern research shows that the use of multilevel modeling methods and effective constitutive models is one of the most promising areas in the description of complex multicomponent materials. The authors of [51] review recent advances and approaches in multiscale models for the further implementation of digital twins. In [52], multiscale modeling is used to describe the behavior of 3D-printed continuous composites. The authors of [53] propose a multiscale modeling approach suitable for modeling complex dynamic reactions and fracture mechanisms.

**Figure 2:**
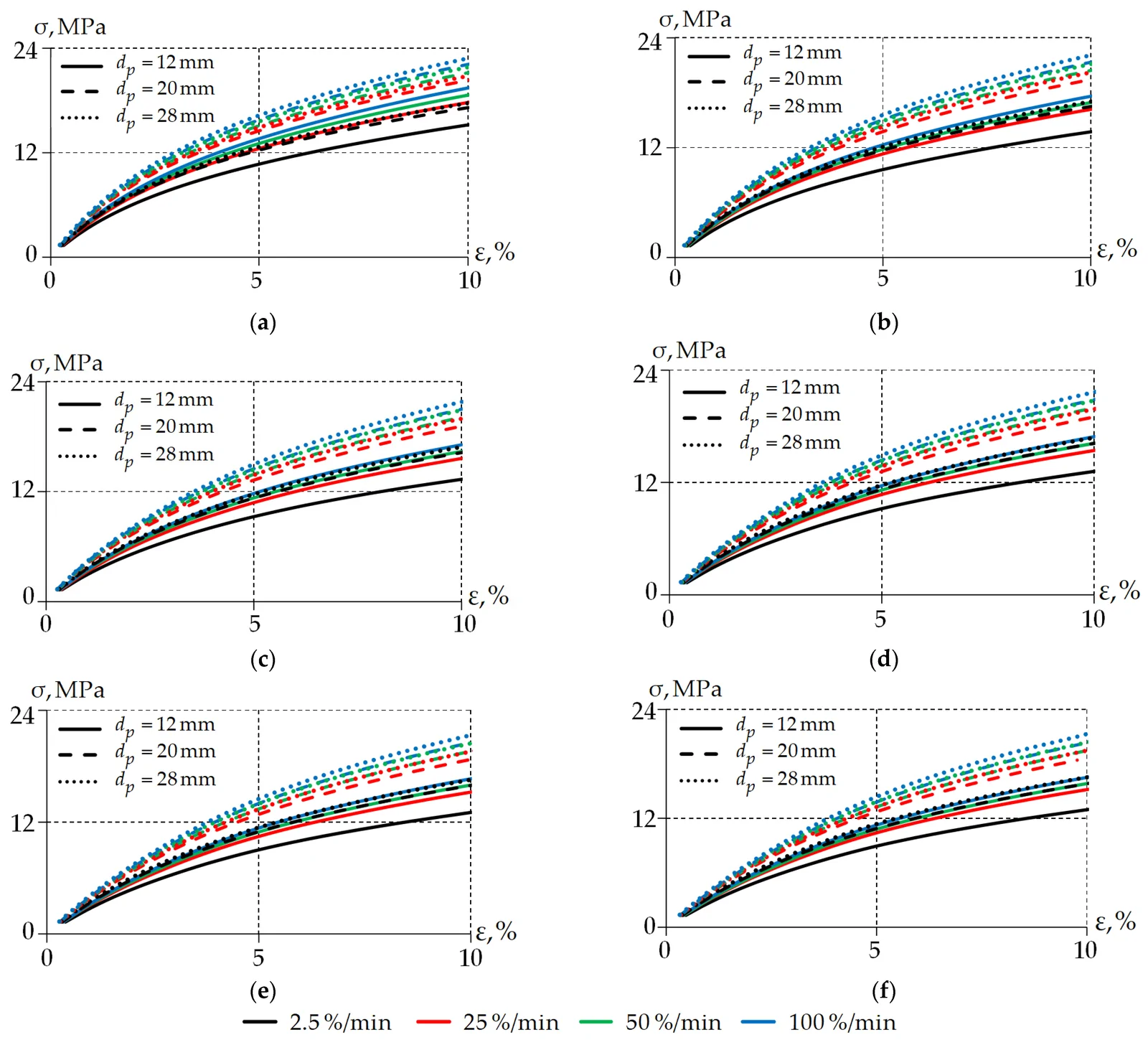
Stress dependence on strain at different strain rates: (**a**) is 233 K; (**b**) is 253 K; (**c**) is 273 K; (**d**) is 293 K; (**e**) is 323 K; (**f**) is 353 K.

Figure 2 shows the calculated stress–strain diagrams obtained at various temperatures, deformation rates, and diameters of the design volume. Each diagram corresponds to the uniaxial loading of the design volume and reflects the combined response of polymer and grease materials.

Stress reduction with the same amount of deformation, regardless of the loading rate, is observed with increasing temperature. This behavior is associated with a decrease in the stiffness of the combined material. A similar thermal softening is observed in pure polymers with increasing temperature and is associated with molecular segmental motion [54]. According to [55], amorphous materials are also prone to softening during “adiabatic” heating, associated with the deformation of samples as a result of inelastic dissipation. This leads to a more intensive development of viscoplastic deformations.

A pronounced stress dependence on the rate of deformation was observed for each of the temperatures considered. Similar trends were established by Zhang et al. [56], where pure UHMWPE demonstrated strong viscoelastic plasticity through three stages of deformation: linear elasticity, fluidity, and plasticity. The stress level increased over the entire strain range with an increase in the strain rate from 2.5 to 100%/min, and was comparable to that of the pure UHMWPE studied in [56]. The temperature effect had the opposite direction. When the deformation rate is fixed, an increase in temperature leads to a decrease in stress.

The results obtained indicate the presence of velocity sensitivity of the combined material. This may be due to the secondary molecular process of polymers and the hardening of the polymer chain with an increase in the strain rate [57,58]. Structural changes inside amorphous materials at high strain rates, including with local heating, are noted in the literature [55]. This suggests the need to take into account the mechanical-thermal coupled deformation in the construction of material structures, including during homogenization.

The stress level also increases as the diameter of the design volume increases. This is due to a change in the effective characteristics of the equivalent medium resulting from the formation of a representative distribution of the grease in the volume of the model.

The obtained data set provides a change in the stress state of the material over a wide range of temperatures and strain rates. This makes it possible to identify the elastic and viscoplastic parameters of the constitutive model. The use of several dimensions of the design volume additionally makes it possible to determine the influence of the geometry of the equivalent medium on the found constitutive parameters. This allows us to consider various ways of filling the polymer sliding layer with recesses for the grease.

In total, 92 types of calculated data were used for identification, which ensures the certainty of the identification problem and increases the stability of the search for model parameters. The effectiveness of using calculated and/or artificial intelligence-generated data in the formation of constitutive models of materials and structures based on experimental data has been confirmed by a number of studies [55,59].

### 3.2. Elastic Characteristics of an Equivalent Medium

**Figure 3:**
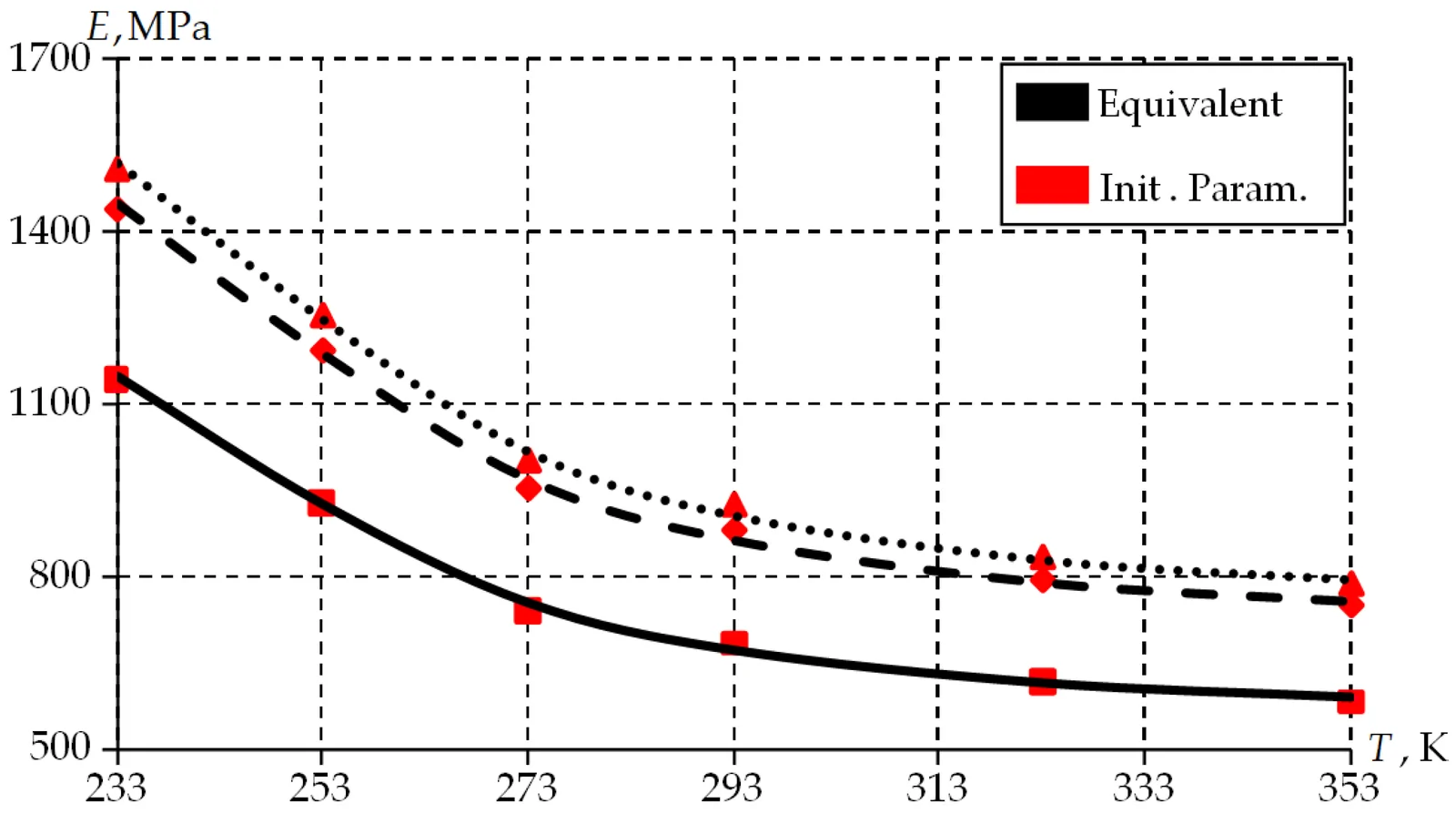
Dependence of the effective Young’s modulus of the design volume on temperature: the solid line is $d_{p}$ = 12 mm; the dashed line is $d_{p}$ = 20 mm; dots are $d_{p}$ = 28 mm; markers are joint deformation of a pair of polymer–grease materials; the square is $d_{p}$ = 12 mm; the rhombus is $d_{p}$ = 20 mm; and the triangle is $d_{p}$ = 28 mm.

**Figure 4:**
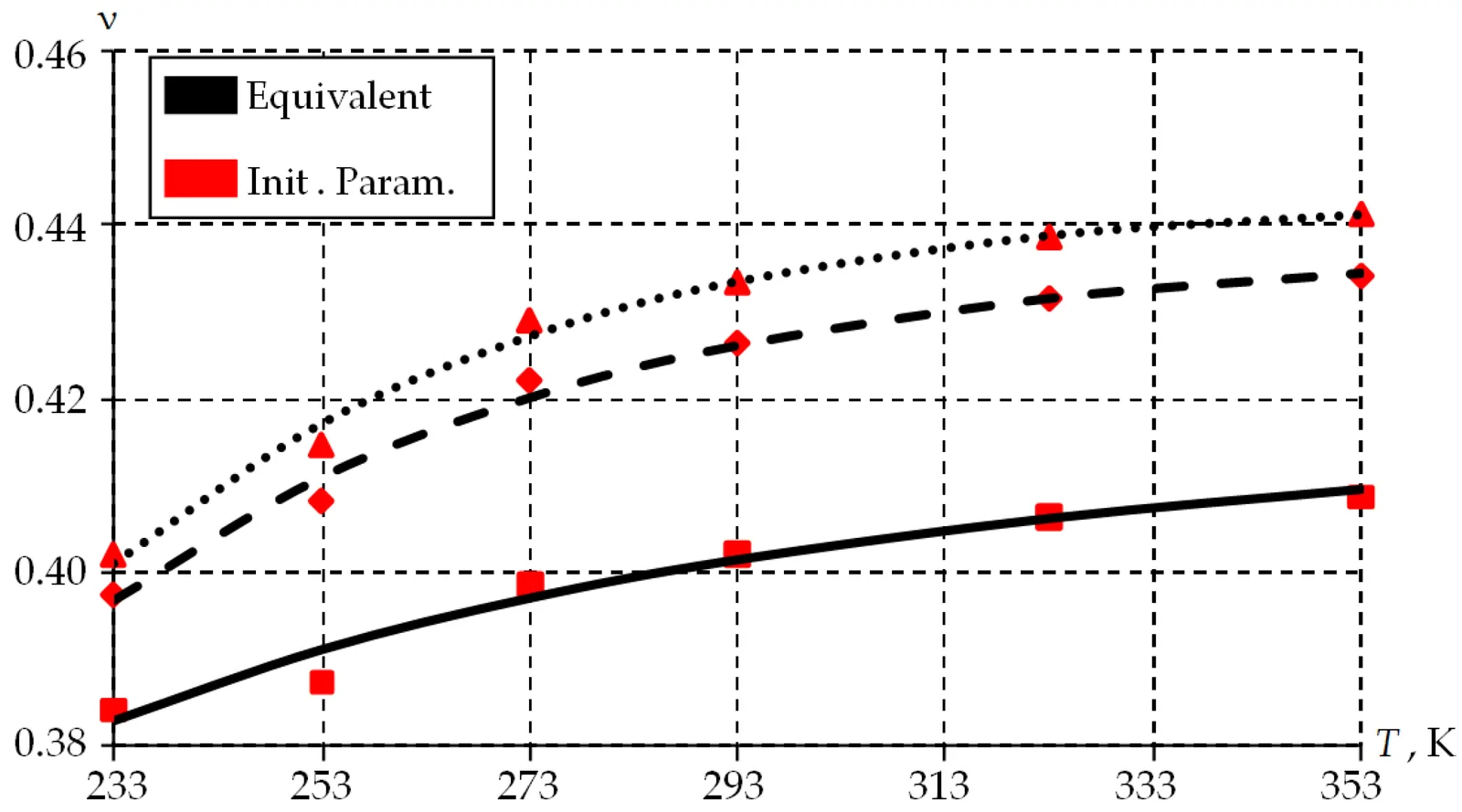
Dependence of the effective Poisson’s ratio of the design volume on temperature: the solid line is $d_{p}$ = 12 mm; the dashed line is $d_{p}$ = 20 mm; dots are $d_{p}$ = 28 mm; markers are joint deformation of a pair of polymer–grease materials; the square is $d_{p}$ = 12 mm; the rhombus is $d_{p}$ = 20 mm; and the triangle is $d_{p}$ = 28 mm.

The effective elastic characteristics of the design volume were determined using the developed model based on the data obtained in Section 3.1. The values of Young’s modulus (Figure 3) and Poisson’s ratio (Figure 4) for the design volume were obtained.

An analysis of the obtained dependencies shows that an increase in temperature leads to a decrease in the effective Young’s modulus for all the studied dimensions of the design volume. When the temperature changes from 233 to 353 K, the decrease in modulus is about 40–45% for a design volume with a diameter of $d_{p}$ 12 mm and 45–50% for a volume with a diameter of $d_{p}$ 28 mm. A similar trend is observed for pure thermoplastic materials with increasing temperature [60].

The effective Young’s modulus was compared with the results obtained using the Mori–Tanaka scheme for spherical inclusions to further evaluate the obtained results [61]. This approach is chosen taking into account the spherical geometry of the region filled with grease. A comparative assessment of the values obtained using FEM ($E_{\mathrm{FEM}}$) and the Mori–Tanaka ($E_{\mathrm{MT}}$) scheme at a temperature of 233K is presented in Table 3.

An increase in the diameter of the calculated volume leads to an increase in the effective Young’s modulus. This is observed in all approaches considered. Mori–Tanaka-based estimates overestimate elastic properties by an average of 30%. This significant discrepancy may arise because the calculated volume only considers a portion of a sphere.

An increase in the diameter of the design volume leads to an increase in the effective Young’s modulus. This is due to a decrease in the influence of boundary effects and an increase in the volume of the material in which a statistically stable distribution of the grease filler is realized. With a small-sized design area, the contribution of the interfacial area becomes significant, which leads to a decrease in effective rigidity.

The dependence of Young’s modulus on temperature increases with an increase in the diameter of the design volume of the antifriction layer with one recess for the grease. This is because the effect of the grease on the overall deformation behavior of the system is reduced. Approximating dependencies with an accuracy of 2% describe the dependence of Young’s modulus on temperature and the diameter of the design volume.

Unlike Young’s modulus, Poisson’s ratio shows an increase with increasing temperature. The rigidity of the polymer material decreases with increasing temperature. This leads to a redistribution of deformations between the components and a change in the transverse deformation reaction. The quantitative increase in Poisson’s ratio with a temperature change from 233 to 353 K is about 5–8% for all the studied sizes of the design volume. The highest values of Poisson’s ratio are observed for the design volume with a diameter of $d_{p}$ 28 mm. This is due to an increase in the contribution of the polymer component to the formation of the deformation response of the equivalent medium.

The obtained temperature dependencies of Young’s modulus and Poisson’s ratio are used as elastic parameters for further identification and numerical implementation of the constitutive Anand model. Taking into account the change in effective elastic characteristics allows us to correctly describe the change in the stiffness of an equivalent medium under different temperature conditions.

Thus, the obtained dependencies show that the effective elastic characteristics of the design volume are functions of temperature and the size of the simulated area. An increase in the diameter of the design volume leads to a decrease in the influence of local inhomogeneities and stabilization of the obtained values of Young’s modulus and Poisson’s ratio.

### 3.3. Identification of the Constitutive Anand Model Parameters

The Anand constitutive model parameters are found based on the initial stress–strain distributions at different deformation rates and temperatures.

**Figure 5:**
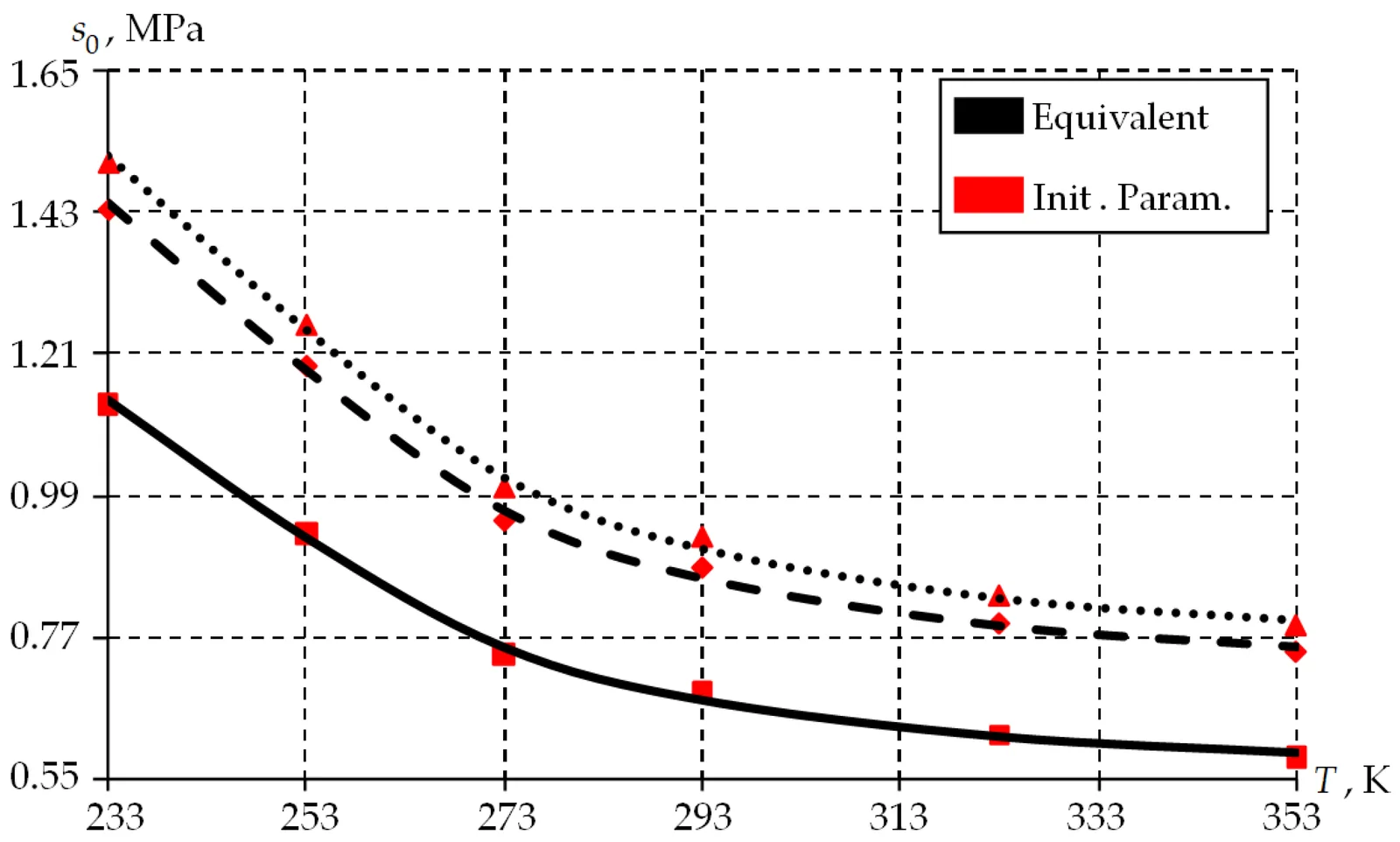
Temperature dependence of the initial value of the deformation resistance parameter: the solid line is = 12 mm; the dashed line is $d_{p}$ = 20 mm; dots are $d_{p}$ = 28 mm; markers are joint deformation of a pair $d_{p}$ of polymer–grease materials; the square is $d_{p}$ = 12 mm; the rhombus is $d_{p}$ = 20 mm; and the triangle is $d_{p}$ = 28 mm.

The first step in identifying the parameters of the Anand model is the definition of $s_{0}$ (Figure 5). $s_{0}$ characterizes the initial ability of the material to resist the development of a viscoplastic flow. This parameter has a direct effect on the stress level at the beginning of deformation and determines the position of the initial stress–strain curve.

The ratio (20) with an accuracy of 2% describes the dependence $s_{0}$ on temperature for different diameters of the design volume. An increase $s_{0}$ over the entire operating temperature range is observed with an increase in the diameter of the design volume. An increase in the diameter of the design volume leads to an increase in $s_{0}$. This is due to a decrease in the influence of local structural inhomogeneities and a more complete consideration of the contribution of lubrication to the formation of effective deformation resistance.

An increase in temperature leads to a decrease in the initial value of deformation. Similar trends toward a decrease in the physical–mechanical parameters of material fluidity with increasing temperature can be observed when describing the nonlinear behavior of pure UHMWPE [58] and other thermoplastics [62]. This corresponds to a decrease in the resistance of the material to the development of viscoplastic flow during heating. This behavior is due to an increase in the mobility of macromolecular chains of the polymer component and a decrease in intermolecular interaction with increasing temperature [58]. The stress required to start the processes of viscoplastic deformation decreases as a result.

**Figure 6:**
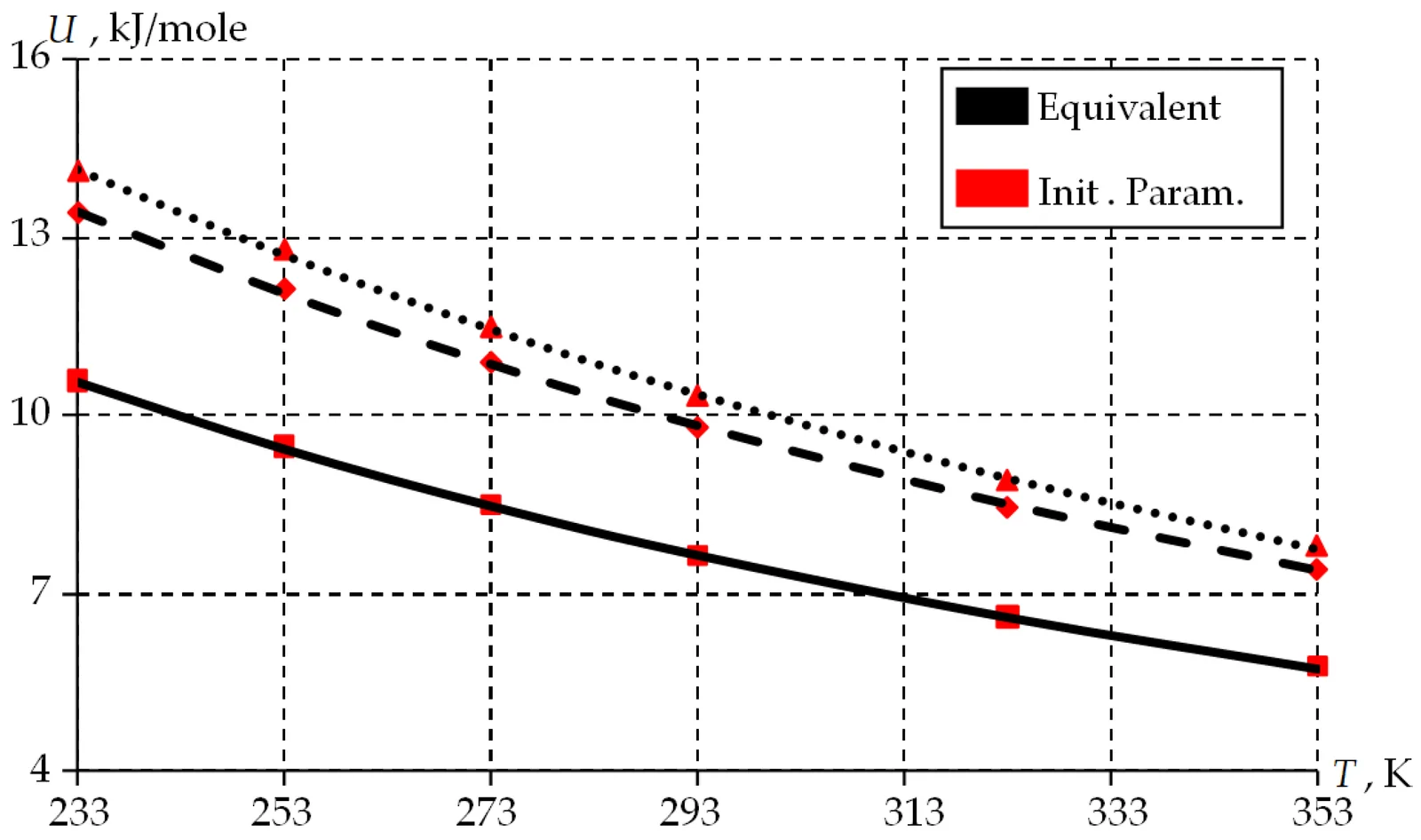
Temperature dependence of the activation energy parameter: the solid line is $d_{p}$ = 12 mm; the dashed line is $d_{p}$ = 20 mm; dots are $d_{p}$ = 28 mm; markers are joint deformation of a pair of polymer–grease materials; the square is $d_{p}$ = 12 mm; the rhombus is $d_{p}$ = 20 mm; and the triangle is $d_{p}$ = 28 mm.

The dependence of *U* on the temperature and diameter of the design volume is shown in Figure 6. *U* characterizes the temperature sensitivity of the processes of viscoplastic deformation of the material. In the Anand model, this parameter determines the effect of temperature on the rate of development of inelastic deformations through the exponential Arrhenius multiplier.

The ratio (21) with an accuracy of 1.5% describes the dependence U on temperature for different diameters of the design volume. An increase in the diameter of the design volume leads to an increase in activation energy.

An increase in temperature leads to a decrease in the activation energy parameter. This indicates a decrease in the energy barrier for the development of a viscoplastic flow when the material is heated. A decrease in the activation energy is associated with an increase in the mobility of polymer chains and a weakening of the intermolecular interaction with increasing temperature. This facilitates the restructuring of the material structure and, as a result, reduces the resistance to viscoplastic deformation.

**Figure 7:**
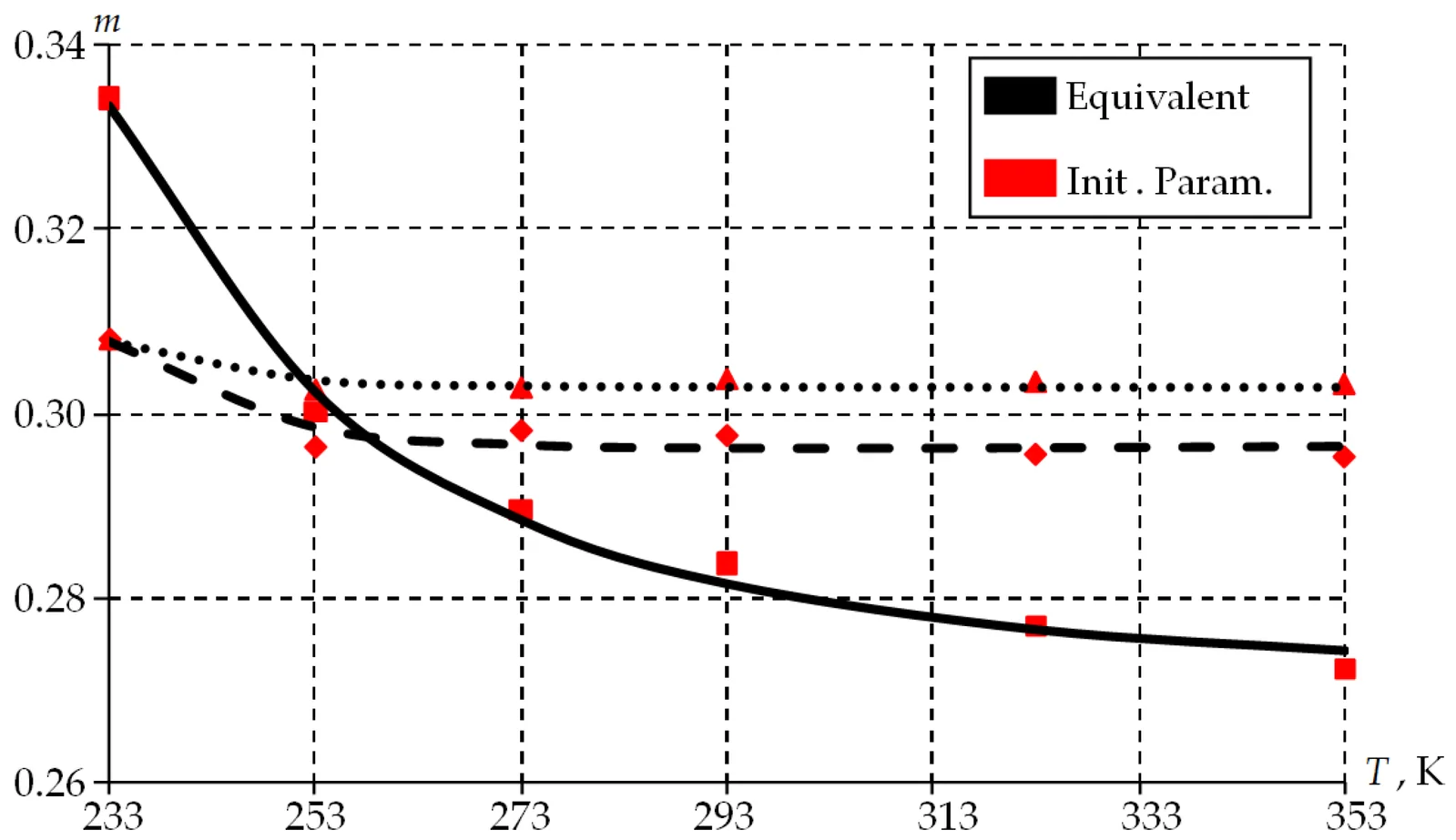
Dependence of the stress rate sensitivity of the stress parameter on temperature: the solid line is $d_{p}$ = 12 mm; the dashed line is $d_{p}$ = 20 mm; dots are $d_{p}$ = 28 mm; markers are joint deformation of a pair of polymer–grease materials; the square is $d_{p}$ = 12 mm; the rhombus is $d_{p}$ = 20 mm; and the triangle is $d_{p}$ = 28 mm.

The dependence of *m* on the temperature and diameter of the design volume is shown in Figure 7. *m* characterizes the sensitivity of the material to the rate of deformation. In the Anand model, this parameter determines the degree of influence of the deformation rate on the viscoplastic resistance of the material and is included in the indicator of the degree of hyperbolic sine, which determines the rate of development of inelastic deformations.

The parameter *m* shows a different pattern of change depending on the size of the design volume. A marked decrease in the value of *m* with increasing temperature is observed for a small diameter of the design volume. However, the parameter change is less significant for large sizes of the design volume. A decrease in the parameter indicates a decrease in the sensitivity of the material to the deformation rate with increasing temperature. This behavior is associated with an increase in the contribution of viscous deformation mechanisms and a decrease in the influence of velocity effects.

An increase in the size of the design volume during the deformation process leads to a more pronounced dependence of the stress state on the loading rate.

The change in $s_{0}$ and *U* characterizes a temperature decrease in the material’s resistance to deformation. The parameter *m* is determined by the balance between the viscous flow mechanisms and the structural features of the equivalent medium. This affects the temperature dependence.

**Figure 8:**
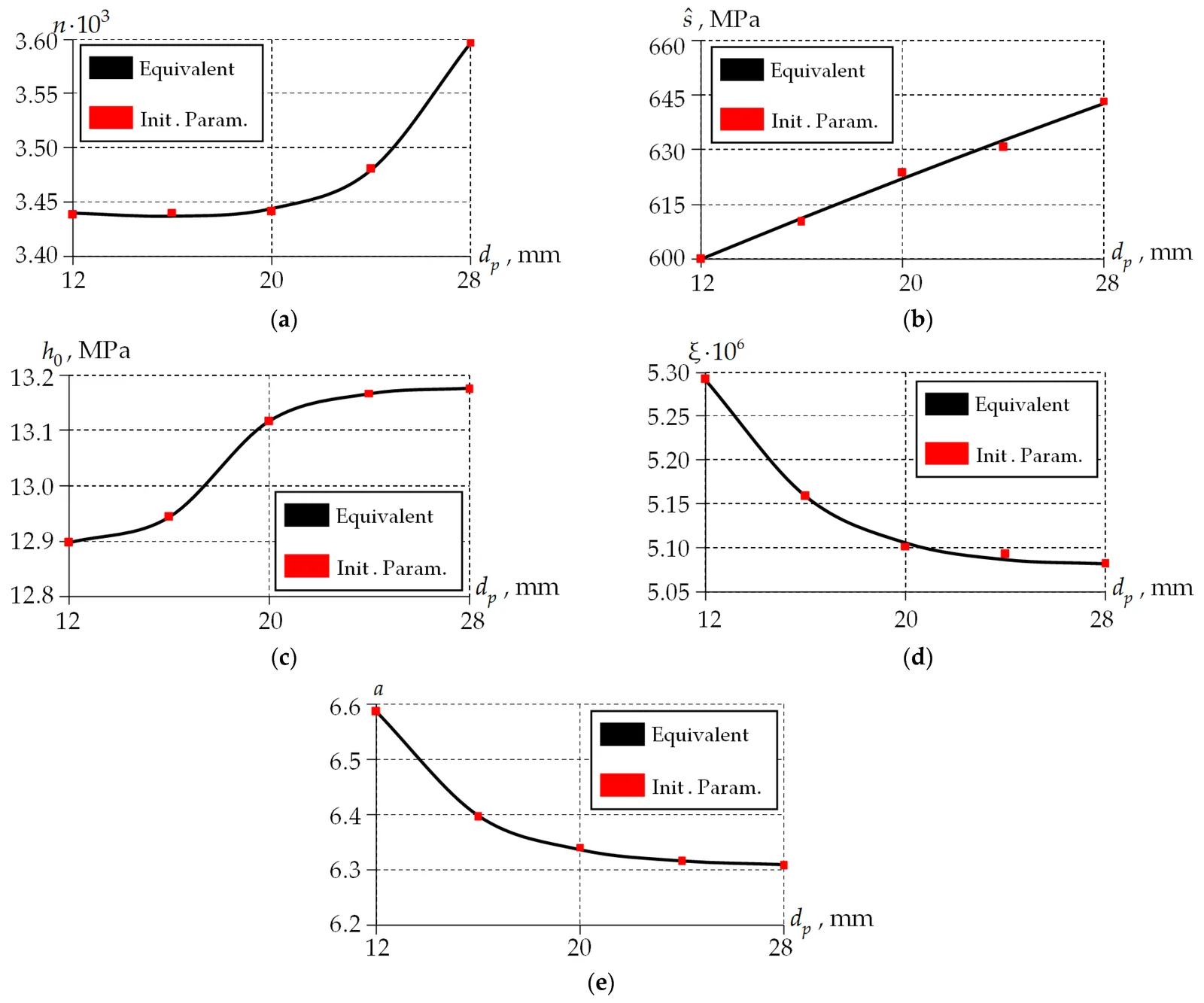
Dependence of the Anand constitutive model A parameters (independent of temperature) on the diameter of the design volume: (**a**) is n; (**b**) is $\hat{s}$; (**c**) is $h_{0}$; (**d**) is ξ; and (**e**) is a; markers are joint deformation of a pair of polymer–grease materials.

The effect of the design volume size on the remaining parameters of the constitutive model was analyzed after determining the temperature dependences of the Anand model’s main parameters (Figure 8). These parameters characterize the processes of hardening, the saturation of the deformation resistance and the nonlinearity of the viscoplastic flow of the material.

The parameter $\hat{s}$ increases with increasing diameter of the design volume. This indicates an increase in the ability of the equivalent medium to resist the development of a viscoplastic flow with an increase in the representative volume of the material. A similar pattern of change is observed for the coefficient $h_{0}$. An increase in this parameter indicates an increase in the material’s ability to harden with an increase in the size of the design volume.

The dependence of the parameter *n* on the diameter of the design volume is non-monotonic. In the range $d_{p}$ = 12–20 mm, the parameter change is insignificant, which indicates a weak effect of the size of the calculated area on the saturation sensitivity of the deformation resistance.

Unlike the parameters $\hat{s}$, $h_{0}$ and *n*, the parameters ξ and *a* decrease with increasing diameter of the design volume. These parameters determine the nonlinearity of the dependence of the strain rate on the stress state and the sensitivity of the material to stress changes. Their decrease indicates a more stable and less localized nature of the viscoplastic flow with an increase in the design volume. The obtained dependencies show that the parameters of the Anand model are scale-dependent characteristics of an efficient environment.

The results show that the effective elastic characteristics of the design volume depend on both the temperature and the size of the homogenization region. An increase in the diameter of the design volume leads to stabilization of Young’s modulus and Poisson’s ratio due to a decrease in the influence of local structural inhomogeneities. This effect indicates the need to choose a representative size of the computational domain when determining effective properties.

A similar relationship is observed for the parameters of the Anand viscoplastic model. The obtained dependencies of the parameters on the diameter of the design volume show that the classical representation of the Anand parameters as constant material characteristics does not always allow us to correctly describe the behavior of a heterogeneous medium. For the contact pair under study, the parameters of the Anand model are effective characteristics reflecting the influence of the heterogeneity of the material.

The mechanical responses of the polymer–grease material structures strongly depend on the deformation rate and temperatures. This confirms the need to take into account the temperature and velocity dependencies of the parameters of material-constitutive models for a qualitative description of the nonlinear behavior of materials, structures, and composite systems [55,62]. However, the current study also highlights the need to take into account the scale-dependent characteristics of composite structures during homogenization.

## 4. Conclusions

A numerical and experimental approach to determining the effective mechanical characteristics of a polymer–grease contact pair based on the representation of the system as an equivalent continuous medium was proposed and implemented in this paper.

The most significant results were as follows.

1. Calculated stress–strain relationships were obtained for various temperatures, strain rates, and computational volume sizes. These relationships take into account a set of experimental data on the physical, mechanical, and thermomechanical properties of the polymer–grease system components.
2. The presence of temperature and velocity sensitivity of the combined material was established. This confirms the need to use a nonlinear constitutive model.
3. The effective elastic characteristics of the equivalent medium were determined. The parameters of the modified Anand model were identified. The model parameters were found to be scale-dependent characteristics of an equivalent medium and to change with a change in the diameter of the design volume.
4. The developed model makes it possible to replace the heterogeneous polymer–grease system with an equivalent medium with effective temperature-dependent and scale-dependent characteristics. This reduces computational complexity when modeling the contact interaction of elements of friction units, such as bridge bearings.
5. The model has a special effect of saving computing resources for multicyclic loading and analyzing the influence of seismic activity on the behavior of the system.

Simultaneous consideration of temperature, strain rate, and the characteristic dimensions of the computational volume allows for the description of the behavior and effective characteristics of a heterogeneous medium with an error of less than 3%. Computational costs are, on average, 11 times lower than those for explicit modeling of the combined deformation of materials.

Despite the results obtained, the approach has a number of limitations.

1. The geometry of the grease recesses is limited by spherical wells. A cylindrical sample with a single recess configuration for the grease is considered. Changing the geometric parameters will change the distribution of the model parameters.
2. The temperature range is limited by the operating conditions in temperate zones. The temperature range is 233 to 353 K. Additional experimental studies in the extended temperature range are required for possible extrapolation of the model.
3. The diameter of the design volume is limited as part of the geometric features of the support part of L-100 (AlfatEch LLC, Perm, Russia) and its spherical and flat sliding layers. The diameter range of the design volume is 12 to 28 mm. Interpolation of the obtained model parameters can lead to a significant deviation from the actual behavior of the design volume.

The main areas of the research development of our scientific group include:

- The development of a three-dimensional model of the design volume, providing for random distribution of the grease.
- An increase in the range of deformation of materials up to 50%.
- Experimental studies on creep and relaxation to verify the obtained parameters on independent data.

## Figures and Tables

**Table 1:** Elastic and viscoplastic parameters of the constitutive model for UHMWPE.

Temp. [K]	233	253	273	293	323	353
*E*, MPa	1831.85	1663.35	1493.83	1343.17	1157.91	1014.48
ν	0.411	0.426	0.441	0.445	0.451	0.453
$S_{0}$, MPa	1.625	1.356	1.083	0.999	0.901	0.851
U, kJ/mole	15.22	13.82	12.42	11.16	9.622	8.430
*m*	0.2928	0.2937	0.2946	0.2946	0.2945	0.2944
*n*	0.00452829					
$\overset{⌢}{s},\;\mathrm{MPa}$	683.85					
ξ	$5.0124\times 10^{-5}$					
$h_{0},\;\mathrm{MPa}$	13,904.13					
a	6.3032					

**Table 2:** Elastic and viscoplastic parameters of the constitutive model for CIATIM-221.

Temp. [K]	233	253	273	293	323	353
*E*, MPa	1095.06	131.56	25.68	13.41	5.59	5.09
ν	0.4999					
$S_{0}$*,* MPa	1.1925	0.5700	0.3501	0.2607	0.2082	0.1886
U,kJ/mole	6.2337	4.6954	3.4263	2.3622	1.0542	0.00277
*m*	1.0365	0.4036	0.1817	0.09247	0.0408	0.0219
*n*	0.26588					
$\overset{⌢}{s},\;\mathrm{MPa}$	0.42039					
ξ	0.01271					
$h_{0},\;\mathrm{MPa}$	6.4759					
a	3.5849					

**Table 3:** Comparative analysis of effective characteristics.

$d_{p}$, mm	12	20	28
$E_{\mathrm{MT}}$, MPa	1736.60	1797.60	1814.30
$E_{\mathrm{FEM}}$, MPa	1141.91	1438.95	1507.62

## Data Availability

The original contributions presented in this study are included in the article. Further inquiries can be directed to the corresponding author.
